# Health worker factors associated with prescribing of artemisinin combination therapy for uncomplicated malaria in rural Tanzania

**DOI:** 10.1186/1475-2875-12-334

**Published:** 2013-09-21

**Authors:** Majige Selemani, Irene M Masanja, Dan Kajungu, Mbaraka Amuri, Mustafa Njozi, Rashid A Khatib, Salim Abdulla, Don de Savigny

**Affiliations:** 1 Ifakara Health Institute, P.O. Box 78373, Dar es Salaam, Tanzania; 2 INDEPTH Network Effectiveness and Safety Studies of Antimalarial in Africa (INESS), Accra, Ghana; 3 Swiss Tropical and Public Health Institute, Socinstrasse 57, CH-4002, Basel, Switzerland; 4 University of Basel, Petersplatz 1, CH-4003, Basel, Switzerland

**Keywords:** Uncomplicated malaria, Correct prescription, Artemisinin combination therapy

## Abstract

**Background:**

Improving malaria case management is partially dependent on health worker compliance with clinical guidelines. This study assessed health worker factors associated with correct anti-malarial prescribing practices at two sites in rural Tanzania.

**Methods:**

Repeated cross-sectional health facility surveys were conducted during high and low malaria transmission seasons in 2010 and collected information on patient consultations and health worker characteristics. Using logistic regression, the study assessed health worker factors associated with correct prescription for uncomplicated malaria defined as prescription of artemisinin-based combination therapy (ACT) for patients with fever and *Plasmodium falciparum* asexual infection based on blood slide or malaria rapid diagnostic test (RDT) according to national treatment guidelines.

**Results:**

The analysis included 685 patients with uncomplicated malaria who were seen in a health facility with ACT in stock, and 71 health workers practicing in 30 health facilities. Overall, 58% of malaria patients were correctly treated with ACT. Health workers with three or more years’ work experience were significantly more likely than others to prescribe correctly (adjusted odds ratio (aOR) 2.9; 95% confidence interval (CI) 1.2-7.1; p = 0.019). Clinical officers (aOR 2.2; 95% CI 1.1-4.5; p = 0.037), and nurse aide or lower cadre (aOR 3.1; 95% CI 1.3-7.1; p = 0.009) were more likely to correctly prescribe ACT than medical officers. Training on ACT use, supervision visits, and availability of job aids were not significantly associated with correct prescription.

**Conclusions:**

Years of working experience and health worker cadre were associated with correct ACT prescription for uncomplicated malaria. Targeted interventions to improve health worker performance are needed to improve overall malaria case management.

## Background

Effective treatment of malaria patients is a cornerstone of global control efforts to reduce malaria morbidity and mortality. However, effective malaria case management depends on early recognition of signs and symptoms as well as the clinical skills of the health worker. In most malaria-endemic areas, management of malaria patients is determined by the clinical presentation and, where possible, laboratory or rapid diagnostic test confirmation [1]. Many countries use evidence-based clinical practice guidelines to help health workers assess, diagnose and treat patients with malaria. To implement treatment guidelines, common strategies are to provide health workers with in-service training, printed copies of the new malaria treatment guidelines, and wall charts and other job aids, as well as providing supportive supervision on a regularly scheduled basis. Despite training, job aids and supervision, health workers have often do not follow recommended guidelines because of shortage of drugs, financial influences, patient load, patient demands, lack of materials and prescribing attitude [2-6]. Different types of treatment error were reported, each with different assumed clinical consequences [7]. For example, treatment with an anti-malarial drug that is effective but not recommended by the guideline has been recognized as a common error in some studies [7]. Other studies have documented that clinical practices can be influenced by factors other than treatment recommendations, such as age of the patient and suspected concurrent conditions [8].

Improving health workers’ compliance with treatment guidelines remains critical to the success of any new drug policy. In Tanzania, as in many sub-Saharan countries, effective implementation of clinical guidelines is a critical challenge, especially for malaria, which is a leading cause of child morbidity and mortality [9,10]. This challenge is particularly relevant today because new policies are being implemented that recommend artemisinin-based combination therapy (ACT), and parasitological confirmation with malaria rapid diagnostic tests (RDT) or blood slide microscopy. ACT is more expensive and has more complex dosing regimens than previously used anti-malarials. However, there are relatively few reports on the quality of clinical practice following implementation of ACT policy in Africa [11,12], particularly those focusing on factors specific to health workers.

The INDEPTH Network Effectiveness and Safety Studies of Anti malarial Drugs in Africa (INESS) implemented health systems studies using the systems effectiveness framework to evaluate ACT in the routine health system. Separate modules were used for assessing each dimension of the framework (access, diagnostic targeting, provider compliance and patient adherence) [13]. As part of this effort, INESS conducts assessments of the quality of malaria case management using a series of health facility surveys to evaluate ACT prescribing patterns among patients with and without parasitologically confirmed uncomplicated malaria. This paper presents findings from an assessment of the quality of malaria case management and health worker factors associated with correct prescription of ACT for management of uncomplicated malaria patients in rural health facilities in Tanzania.

## Methods

### Study area

The study was conducted in Rufiji and Ifakara Health and Demographic Surveillance System (HDSS) sites. Rufiji HDSS is situated in Rufiji District, Coast Region, with a catchment population of approximately 85,000 people living in 16,000 households [14]. Ifakara HDSS is situated in and covers parts of Kilombero and Ulanga Districts in Morogoro Region. Ifakara HDSS site constitutes more than 99,000 people, living in 28,000 scattered rural households [15]. The two HDSS sites have higher malaria transmission during the major rainy season, usually occurring between March and June annually. Malaria transmission in these study areas is endemic with seasonal fluctuations. Malaria parasitaemia is also most prevalent during a period of long rain from March to June.

Health services in Tanzania are provided by the government and non-government organizations; over 70% of health facilities are in rural areas where the majority of the population lives [16]. Levels of health care delivery in Tanzania start with home/village/community primary health care post, pharmacy, and drug stores, including accredited drug dispensing outlets (ADDO), followed by dispensaries, health centres, district hospitals, regional hospitals and the highest level is referral/consultant hospital [16]. The Rufiji HDSS has 24 health facilities in their surveillance area (one non-government hospital, two health centres and 21 dispensaries) while in Ifakara HDSS there are 14 health facilities (two health centres and 12 dispensaries) [13]. A dispensary caters for between 6,000 to 10,000 people and supervises all the village health posts in its ward; a health centre is expected to cater for 50,000 people, which is approximately the population of one administrative division [17]. A dispensary has medical assistant/clinical officers, assistant clinical officers, and lower cadres (nurse or nurse midwife, rural health assistant and nurse aide) while a health centre has a medical assistant/assistant medical officer/clinical officer, an assistant clinical officer and lower cadres. The hospital has a good mix of qualified staff of different specialism and experiences, including graduate medical officers, medical assistant/assistant medical officer/clinical officers, assistant clinical officers and lower cadres [17].

### Sample size and power calculations

Sample size was calculated based on the assumption of 75% of malaria patients are treated with ACT and design effect of 2. The target sample size was 720 patients per HDSS to estimate the population of those with uncomplicated malaria correctly treated with ACT with 10% precision, assuming 20% of all patients present with uncomplicated malaria. All government and non-government health facilities providing outpatient care during the survey within HDSS areas were included (17 in Rufiji and 14 in Ifakara) and 7 health facilities were not operating during the survey. Investigators visited each facility for two to three days and collected information on attending patients. All patients attending for initial illness at the health facility on the days of the survey were eligible. Health workers were following the national guidelines for diagnosis and treatment of malaria.

### Study design and data collection

This was a two sets cluster survey were conducted: one in high malaria transmission season (March, 2010) and another in low malaria transmission season (November 2010), where a cluster was defined as all patient consultations performed in a health facility on the one day of survey during regular working hours. The survey was conducted in all outpatient health facilities licensed to prescribe ACT (artemether-lumefantrine, AL) within the Rufiji and Ifakara HDSS sites.

Twenty interviewers and two supervisors were trained together on survey procedures and blood slide collection in a classroom setting and then practiced these activities in test health facilities which were different from those surveyed in the study. At health facilities, all health workers performing patient consultation on the day of survey were given an identification number. All outpatients presenting for initial consultation on the day of survey, and who consented to participate in the survey, were interviewed prior to leaving the health facility. Patients eligible for the survey were asked to provide informed consent. All patients who consented were included in the survey and given a study identification card with their study identification number. The health workers seeing a consented patient noted their initials and health worker identification number on the patient’s study identification card as well the patient’s medical record (file) number. In the laboratory, the patient identification number on the study identification card was used to label any extra blood slides made for the patient.

When the patient was ready to leave the health facility after visiting the laboratory and pharmacy as needed, the surveyor interviewed the patient and collected a blood smear (if a blood smears had not been done in the laboratory). The interview was used to determine if patients had understood the information provided by the health worker regarding diagnosis, referral, treatment, follow-up, and home care. All information and prescribed medications were recorded on a standardized questionnaire.

At the end of the survey day all health workers who had performed patient consultations were interviewed to collect information on their demographics, cadre, pre-service and in-service training, work experience, access to printed copies of the national malaria treatment guidelines and wall charts, and exposure to supervision in the preceding six months. Finally, a health facility assessment was undertaken to record the availability of AL and other anti-malarials on the survey day and in the previous three months, malaria diagnostics and any displayed case-management wall charts.

### Definition

Uncomplicated malaria in this analysis was defined as either: 1) history of fever in the previous 48 hours; or 2) presence of fever at the time of presentation, both confirmed by blood slide or RDT positive for malaria infection. For children aged two to 59 months, fever or history of fever in the previous 48 hours was also considered a clinical diagnosis of uncomplicated malaria according to integrated management of childhood illness classification, if a blood slide or RDT was unavailable or not done. Correct prescription of ACT was defined as prescription of AL for patients with a qualifying diagnosis of uncomplicated malaria as described above.

### Data management and analysis

Data were double entered into EPIDATA version 3.1 [18] and validated by checking completeness and consistency. The analyses were performed using STATA Version 11.0 [19], using survey procedures that account for clustering and stratification. All percentages and odds ratios reported are population-average estimates which have been adjusted to take into account the clustering of the study design.

The outcome variable in this analysis was patients with uncomplicated malaria correctly prescribed an ACT and the determinant/explanatory variables were health worker factors: health worker cadre, in-service training, having three or more years’ working experience, supervision visit in previous six months, age of health worker, and availability of job aids. In-service training was classified into two groups: first, health workers trained on either malaria case management, integrated management of childhood illness (IMCI) or use of new anti-malarials; and, second, health workers’ who were not trained in any of the above three categories. Availability of job aids included possession of the printed national malaria treatment guideline and wall chart that described the current treatment recommendations. Availability of job aids was assessed by observation by the study team. Ages of health worker were classified into three groups: age below or 35, 36 to 60 and finally health worker with age above 60.

To identify health worker factors associated with correct prescribing practices, treatment practices were analysed at all health facilities with AL on the survey day. The outcome variable in logistic regression model was coded “1” if a patient with uncomplicated malaria was correctly prescribed an ACT and “0” if a patient with uncomplicated malaria had not received a correct prescription of ACT. Health worker factors suspected to be associated with correct prescription of ACT for uncomplicated malaria were identified in the multivariate model.

The analysis used data from both seasons and employed both descriptive and analytical statistics. Frequency distribution of responses by categories of each variable were calculated and presented. Further analysis was performed in multivariable fashion using logistic regression to assess health worker factors associated with correct prescribing of ACT for uncomplicated malaria. Explanatory variables were selected for inclusion in the multivariate logistic regression automatically regardless of the significance status in the univariate analysis because all explanatory variables are of interest. The model was checked for statistical interactions and adequacy before being approved as final. An alpha level of 0.05 was used for all tests of significance. The study did not consider children aged below two months or children weighing below five. Because the guideline for management of malaria indicates children weighing below five kilogram or age below 2 months are not recommended for AL [1].

### Ethical approval

Ethical approval for this study was received from the Ifakara Health Institute Ethical Review Board (IHI/IRB/No.A67-2009) and national ethical clearance.

## Results

### Patient characteristics

**Figure 1:**
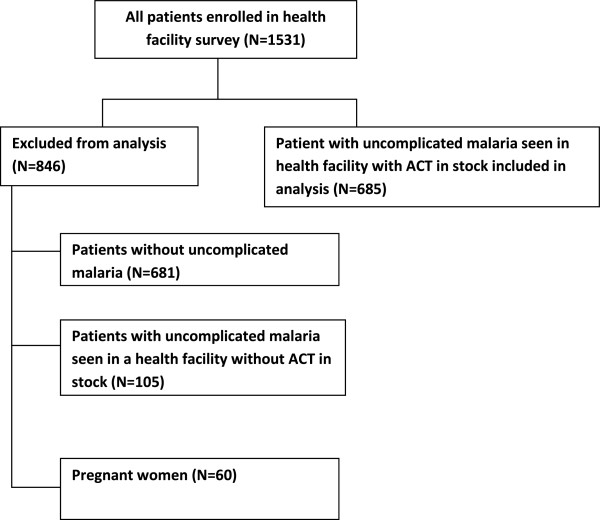
Inclusion of patients in the analysis.

Figure 1 presents details of patients included in analysis. Data were collected from 31 health facilities and analysis is based on 685 patients with uncomplicated malaria seen in 30 health facilities with ACT in stock on the day of survey. A total of 846 patients were excluded from analysis. Pregnant women (N = 60), patients without uncomplicated malaria (N = 681) and patients with uncomplicated malaria seen in a health facility without ACT in stock (N = 105) were excluded from the analysis (Figure 1). More than half (54%) of the patients with uncomplicated malaria were children under five and 55% were females (Table 1). Thirty-four per cent were diagnosed by either microscopy or RDT while 66% were clinically diagnosed.

**Table 1:** Patient characteristics and ACT prescription by patients characteristics included in analysis

**Patient demographic**	**n/N**	**%**	**95%CI**
**Patient age**			
Mean age in years (range)	11 (0–80)		
Children under 5 years	370/685	54.0	50.3-57.8
5 years and above	315/685	46.0	42.3-49.7
**Patient gender**			
Female	373/685	54.5	51.0-57.9
Male	312/685	45.5	42.2-49.0
**Type of malaria diagnostic**			
Patient diagnosed by microscopy	115/685	16.8	10.8-25.2
Patient diagnosed by mRDT	114/685	16.6	11.1-24.3
Patient diagnosed clinically	456/685	66.6	56.6-75.2
**Patient treatment**			
Patient prescribed ACT	398/685	58.1	51.9-64.1
**Patient prescribed ACT by age**			
Under 5	219/370	59.2	51.9-66.1
5 and above	179/315	56.8	49.6-63.8
**Patient prescribed ACT by type of diagnostic**			
Diagnosed by microscopy	70/115	60.9	48.5-72.0
Diagnosed by mRDT	95/114	83.3	72.9-90.3
Clinically diagnosed	233/456	51.1	42.8-59.4
**Patient prescribed ACT by health worker cadre**			
Seen by medical officer	13/32	40.6	27.8-54.9
Seen by clinical officer	276/488	50.6	48.6-64.2
Seen by nurse and lower cadre	109/165	66.1	56.5-74.5
**Patient prescribed with Non-ACT**	287/687	41.9	35.9-48.2
SP	5/685	0.7	0.3-2.1
Quinine	71/685	10.4	7.4-14.3
Other anti-malarial	1/685	0.1	0.02-1.1
Not prescribed with any anti-malarial	210/685	30.7	27.2-34.3

### Malaria treatment received by patients

Overall, just over half (58%) of patients with uncomplicated malaria seen in health facilities with ACT in stock received an ACT (Table 1). Quinine, a second-line treatment for uncomplicated malaria was given to 10% of all patients. Only 1% received sulphadoxine-pyrimethamine (SP) in place of the first-line treatment and 31% of malaria patients received no anti-malarial drug. Sixty six percent of patients seen by nurse and lower cadres were prescribed by ACT and 41% seen by medical officer were prescribed ACT. The national malaria treatment guidelines recommend presumptive treatment of a fever with ACT for children under five who cannot be closely monitored and when malaria tests are not available, only half (51%) of patients who were clinically diagnosed were prescribed ACT (Table 1).

### Health facility and health worker characteristics

Few health facilities had both malaria diagnostic microscopy and RDT (Table 2). At the time of the survey, field staff verified that ACT was in stock in 94% of health facilities surveyed. Seventy-one per cent of the patients with uncomplicated malaria seen in health facility with ACT in stock were seen by a medical assistant, clinical officer or clinical assistant, only 5% of patients were seen by a medical officer and 67% were seen by health workers who had received in-service training. Seventy-seven per cent of patients were seen by a health worker with three or more years’ working experience. Twenty-two per cent of patients were seen by health workers who had received at least one supervision visit in the previous six months and 42% of patients were seen by health workers who had job aids in possession. While 19.6% patients were seen by health workers age below or 35, 75% were seen by health workers aged 36 to 60 and only 5.4% were seen by health worker aged above 60 (Table 2).

**Table 2:** Health facility and health worker characteristics

	**n/N**	**%**	**95%CI**
**Availability of malaria diagnostic**			
HF with microscopy	6/30	20.0	7.7-38.6
HF with mRDT	18/30	60.0	40.6-77.3
HF with both microscopy and mRDT	6/30	20.0	7.7-38.6
**Health workers**			
Medical officer	6/71	8.4	3.2-27.5
Clinical officer	40/71	56.3	44.0-68.1
Nurse aide and lower cadre	25/71	35.2	24.2-47.5
**Patient seen by health worker(Age)**			
Patient seen by Health workers with age below and equal 35	134/685	19.6	12.3-29.8
Patient seen by health workers with age 35-60	514/685	75.0	64.9-83.0
Patient seen by health workers with age above 60	37/685	5.4	2.6-11.0
**Patient seen by health worker (cadre, experience, training and job aids)**			
Patient seen by a medical officer	32/685	4.7	2.4-9.0
Patient seen by a clinical officer	488/685	71.2	60.5-80.0
Patient seen by nurse aide or lower cadre	165/685	24.1	15.9-34.8
Patient seen by HW with 3 or more years’ experience	525/685	76.6	68.0-83.5
Patient seen by HW who had supervision visit in the previous 6 month	153/685	22.3	15.0-32.0
Patient seen by HW who received in-service training in malaria case management*	458/685	66.9	55.4-76.6
Patient seen by HW with malaria case management job aid**	289/685	42.2	30.7-54.5

*In-service training includes malaria case management, integrated management of childhood illness (IMCI), or use of new anti-malarials.

**Job aids include possession of the national malaria guideline and wall chart.

### Health worker factors associated with correct prescription of ACT

Table 3 presents factors health worker factors associated with correct prescription of ACT. Patients seen by a clinical officer and nurse or lower cadre health worker had significantly higher odds of correctly getting an ACT than patients seen by a medical officer. Health workers with three or more years’ work experience had significantly higher odds of correctly prescribing ACT (adjusted OR = 2.9, 95%CI: 1.2-7.1). In-service training, supervision visit and availability of job aids for health workers were not associated with prescribing ACT.

**Table 3:** Health worker factors associated with correct prescription of ACT (N = 685)

	**Univariate**		**Multivariate**	
**Variable**	**Unadjusted odds ratio (95% CI)**	**P-value**	**Adjusted odds ratio (95% CI)**	**P-value**
**Health worker cadre**				
Seen by medical officer	Ref		Ref	
Seen by a clinical officer	1.9(0.99-3.63)	0.051	2.2(1.05-4.49)	0.037
Seen by a nurse aide or lower cadre	2.8(1.41-5.73)	0.004	3.1(1.34-7.12)	0.009
**Working experience,**				
Seen by HW with less than 3 years work experience	Ref		Ref	
Seen by HW with 3 or more years’ work experience	2.7(1.30-5.65)	0.008	2.9(1.20-7.10)	0.019
**Supervision visit**				
Seen by HW who had no supervision visit	Ref		Ref	
Seen by HW who had supervision visit in the previous 6 months	0.8(0.42-1.48)	0.460	1.3(0.55-3.22)	0.515
**In-service training**				
Seen by HW who not received in-service training	Ref		Ref	
Seen by HW who received in-service training in case management	1.3(0.77-2.09)	0.343	1.1(0.57-1.83)	0.942
**Job aids**				
Seen by HW with no malaria case management job aid	Ref		Ref	
Seen by HW with malaria case management job aid	1.3(0.79-2.09)	0.313	1.1(0.63-1.83)	0.788
**Age of health worker**				
Seen by HW aged below 35 or 35	Ref		Ref	
Seen by HW aged 36-60	1.3(0.68-2.35)	0.450	0.9(0.47-1.78)	0.791
Seen by HW aged above 60	1.0(0.46-2.25)	0.974	1.0(0.49-2.15)	0.948

## Discussion

This study aimed to assess a limited range of health worker factors possibly associated with correct ACT prescribing practices at health facilities. The findings provide some explanations to help understand predictors of correct prescription of ACT for uncomplicated malaria. ACT was more commonly prescribed by clinical officers, nurse aides or lower cadres than by medical officers. This finding was similar to that observed during the era of SP policy in Kenya [20] and chloroquine policy in Benin [6]. Findings agreed with findings from the study by Zurovac *et al.* in Kenya in which nursing aides who are considered less trained compliance much more closely to treatment guidelines than clinical officer [20]. Several reasons may explain these results. First, there are always more lower level cadres health workers (clinical officers, nursing aide and lower cadre) in rural health facilities who interface with patients with common illnesses compared to doctors or medical officers. This is because the health sector is facing a serious human resource crisis that negatively affects the ability of the sector to deliver quality services. There is a severe shortage of human resources at all levels, particularly in rural districts [21]. Medical officers are more frequently involved in administrative work and at hospital level where referral cases are expected. For malaria, this could mean patients who did not respond well to first line treatment at lower level of care i.e. dispensary or health center hence sent to hospitals for further management. This may explain the significant findings of medical officer for prescribing other than ACT for patients with uncomplicated malaria.

Second, clinical officers and nursing aides or lower cadres may be better at following clinical algorithms because they have less of a ‘medical vocabulary’ of alternative diagnoses and treatments that might interfere with the simple ‘fever (and a positive test) = malaria’ concept upon which the guideline is based. Third, medical officers and, to a lesser extent, clinical officers, may have been taught that their clinical judgment can and ought to override guidelines. Indeed, they may view guidelines as suggestions, rather than rules that should be followed systematically. Furthermore, findings reveal that clinical officers and nurse aides or lower cadres commonly perform consultations and follow recommended guidelines than medical officers in rural areas of the formal health system, as has been reported in other settings [20].

Effective ACT prescribing for uncomplicated malaria was considerably below the Roll Back Malaria (RBM) target of 80% of uncomplicated malaria treated with ACT. The study conducted by Ucakacon *et al.* in Uganda in which proportion of prescriptions conforming to Uganda treatment policy was higher at 88% of uncomplicated malaria being prescribed AL [22]. In Kenya and Zambia poor quality treatment practices were observed at public and mission facilities soon after ACT was introduced as first-line treatment, though subsequent studies, up to five years later, showed improvements in the proportion of patients that were prescribed and received ACT [11,12,23]. As found elsewhere, the proportion of patients that were prescribed or received an ACT seems low given the broad availability of ACT at health facilities [11,12,23-26]. Hence, there is a great need to improve the quality of malaria case management for uncomplicated malaria in rural Tanzania, with a greater emphasis on health worker compliance with clinical guidelines.

Health workers who had three or more years’ working experience were significantly more likely to correctly prescribe ACT in health facilities with ACT than health workers with less than three years’ working experience. This finding however contrast with from study by Ucakacon *et al.* in Uganda in which health workers who had been in service for a shorter time period were more likely to conform to the policy as those who had been in service longer [22]. Several reasons may explain why having more working experience was associated with correct prescribing of ACT. The first is related to health workers’ training. A health worker experienced in caring for patients may have been more likely to be trained on malaria case management, which includes the use of new anti-malarials, and more likely to prescribe correct ACT of interest during the survey. Indeed, theoretically, the working experience might improve health worker performance, but still might have had enough impact to overcome differences between well-performing health workers who were not having three or more years of caring patients and poorly performing health workers with less three years of working experience. Secondly, perhaps through years of experience they came to trust the recommended medicine for management of uncomplicated malaria after having seen patients’ good clinical response following its use. They may also have been more likely to be aware of the prevailing resistance to previously used anti-malarial drugs. Finally they might have had several mentoring visits in the course of their practice, and hence more confidence in prescribing drugs.

The lack of association of in-service training and correct prescribing of ACT is consistent with other studies [6,27-29] and there are several possible explanations. First, the quality of training may have been inadequate. Although the study has no evaluation of the courses, may be they used adult teaching methods and were mostly of an inadequate duration. Perhaps the short time dedicated to clinical practice diminished the training effectiveness. Secondly, health workers who were trained in-service may have been too few to detect an important difference. However, this explanation is unlikely because the estimated odds ratio was substantially higher than 1. A third possible explanation for the lack of association between in-service training and correct prescribing is that training improves knowledge, but knowledge is not sufficient on its own to change clinical performance. The study failed to find an association between in-service training and correct prescribing of ACT is consistent with other studies showing that correct knowledge does not necessarily translate into correct behaviour [28,30]. In the study conducted in Kenya on health worker performance found that in-service training alone may not be adequate to improve health worker prescribing, inclusion of supervision and post-training follow-up should be considered in clinical practice change initiative [29]. Finally, better prescribing of ACT practices could not be attributed to any routine ACT implementation activities (in-service training, guidelines, wall charts). The reviews of previous studies with similar design, as well as systematic reviews of other interventional trials on the use of medicines in developing countries, commonly reported a mixed association between in-service training and health worker performance, and sometimes, as in this study, no association was found with in-service training [8,31].

### Limitations

This study had several important limitations. First, the method of directly observing health workers may have influenced their performance either making them anxious or motivating them to perform better than usual. As one study found, most health workers reported the presence of observers either had a small positive effect or had no effect on their performance [31] since the study used field workers who were local residents of the study areas, the observer’s bias would be minimal. Second, the study had only limited details of the training of health workers on the new treatment guidelines, so could not qualitatively describe how it might have affected correct use of ACT. Third, the evaluation of the quality of supervision was not very detailed. There is a need to conduct further studies to assess other aspects of the malaria treatment like dosage prescribed by health workers and proper drug administration.

## Conclusions

Health workers with three or more years’ experience caring for malaria patients and lower cadre were significant predictors of correct ACT prescription. Although in-service training, supervision visit and availability of job aids did not seem to improve correct use of ACT, we do not imply that training, supervision visit and job aids are unimportant components of the health worker performance, but rather are concerned by the scarcity of evidence from well-designed studies that supports these commonly used interventions. Targeted interventions to improve health worker performance need to be developed and tested to improve overall malaria case management. Interventions above and beyond in-service training, supervision, job aids, etc. will need to be tailored to the specific needs of newly qualified providers (less than three years’ working experience) and medical doctors. Improving malaria case management will be required to reach Roll Back Malaria goal of “near zero” malaria deaths by 2015.

## Competing interests

The authors declare that they have no competing interests.

## Authors’ contributions

MS wrote the manuscript first draft, MIM supervised fieldwork, revised the paper and contributed to the discussion. MS, MN and DK analysed data, revised manuscript and contributed to the discussion. MA, RK, SA and DS revised the paper and contributed to discussion. All authors read and approved the final manuscript.
